# Supplementary material for: Learning curve and surgical outcome of robotic assisted colorectal surgery with ERAS program
Source: Sci Rep. 2022 Nov 29;12:20566. doi: 10.1038/s41598-022-24665-w (PMC9709162; doi:10.1038/s41598-022-24665-w)
Supplement: Supplementary file 1 — Supplementary Information 1. [file 41598_2022_24665_MOESM1_ESM.zip › LSC Raw data-Ñ[▒K/060/H_ñw╝╨░O▒Kñσ.pdf]

正式摘要(電子簽章：00000184299670)

W52 / W52 英文標題(注意:無英文姓名-請補病人基本檔!) 【讀取】憑證

臺中榮民總醫院

出院病歷摘要

轉入號： 轉出號：

[redacted]

(8)流水編號	
(9)入院日期	109/01/08 (2020) 直腸外科 W52 - 162
(10)轉科日期	
(11)出院日期	109/01/15 (2020) 住院天數計 7 日

診斷

- 入院 :Ca of Transver colon
- 出院 :Ca of Transver colon s/p laparoscopic Right hemicolectomy on 2020/01/09

主訴

- occult blood positive in health examination

病史

- This is a 65 year old female patient with history of

  1. Polio s/p bilateral lower extremitties arthroplasty

  2. s/p CS twice

  According to the patient, she incidentaly found occult blood during the health examination. She came to our CRS OPD due to above and recieved colonoscopy which confirmed of T colon cancer. This time, she was admitted to our ward for schedule surgery.

體檢發現

-
  - General appearance :
    - easy looking , conscious clear , without cardiopulmonary distress
    - Vital signs自動帶入date 體溫(BT) 脈搏(P) 呼吸(R) 血壓(BP) 身高(BH) 體重(BW) BMI
      20200108 37.1℃ 82次/分 16次/分 153/65144mmHg 144cm 50kg 24.1
  - HEENT :
    - Eyes: no anemic conjunctiva, no icteric sclera, normal EOM
    - ENT: no otorrhea, no rhinorrhea, no erythematous mucosa, no injected tonsil, no deformity
  - Neck: supple, freely movable, trachea midline,no deformity, no engorged jugular vein , no palpable thyroid or lymph node enlargement
  - Chest:
    - Inspection: Grossly normal, symmetric and fully expansion, no accessory muscle used, no operation scar, no spider angiomata, no gynecomastia
    - Palpation: no palpable mass or nodule, no subcutaneous emphysema, no tenderness
    - Ausculation: clear breathing sound
  - Heart:
    - Percussion: normal heart size
    - Ausculation: regular heart beat, normal S1, S2, no gallop, no murmur
  - Abdomen:
    - Inspection: in
      - flat contour, symetrically, no scar, no stria, no dilated veins, no rashes
    - Ausculation: normoactive bowel sound, no abdominal bruits
    - palpation: soft, no muscle guarding or rigidity, no local tenderness, no rebounding pain, no palpable mass, no palpable pulsation
    - Percussion: no shifting dulllness, no hepatomegaly, no splenomagaly
  -

Peripheral pulsation :	CA	BA	RA	FA	PA	PTA	DPA
Right side :	++	++	++	++	++	++	++/+++
Left side :	++	++	++	++	++	++	++/+++

  - Genito-anus: no tenderness or palpable mass lesion or rectal shelf, normal external genitalia, normal anal tone, brown stool

CRS CONDITION

  -
    - Lung

手術日期及方法

- 手術日期及方法20200109 1. Laparoscopic right hemicolectomy with side-to-side anastomosis, antiperstalsis, GIA 75/3.5*2, re-inforcement with silk (+).
  2. Vessel ligation : ileo-colic, right colic, and right branch of middle colic vessels.
  3. Cut margin: proximal (ileum) 10 cm, (colon) 6 cm, distal 13 cm.
  4. Repair of mesentery defect: nil.
  5. A 12Fr. penrose drain placed at anastomosis site.

住院治療經過

- After the admission, the patient received surgery on 2020/01/09 and tolerated it well. due to her relative stable condition, the patient was discharged on 2020/01/15

合併症

- NIL

一般檢查

- 累積報告：

DATE	TIME	ALB	TP	BIL.T	BIL.D	ALKP	AST	ALT	LDH	CHOL	TG	HDL-C	LDL	UA	NH3	AMY
1090109	1527	3.1	5.1			41	18	14		197	95		136	4.2		
1090113	0915	3.2		0.28			17	12								

DATE	TIME	WBC	RBC	HGB	HCT	MCV	PLT	BLST	NEUT	BAND	SEG	LYM	MON	EOS	BAS
1090109	1527	2830	3.60	10.2	31.3		206		40.6			50.2	6.7	2.1	0.4
1090113	0853	6750	4.19	11.9	36.8	87.8	279		77.6			15.7	5.2	1.2	0.3

DATE	TIME	NA	K	CL	CA	BUN	CREAT	Mg	P	CRP	ESR	PCT	LIPASE	LACTATE
1090109	1527					9	0.37			0.072				
1090113	0915	141	3.3			9	0.49							

DATE	TIME	GLUCOSE	PAB	TRF	ZN	CU	KETONE	CK	CKMB	PT-P	PT-C	APTT-P	APTT-C	TRO-T
1090109	1527	233												

- 放射線報告：檢驗項目 **:CT ABDOMEN-SURVEY** 報告日期 **:1090109**

主　訴：
Result:
Finding:
Imaging Report Form for Colorectal Carcinoma (AJCC 8th)
1. Imaging Date: 20200108
Imaging Modality, Imaging by
CT scan
Imaging Protocol:
Post IV contrast CT scan of the whole abdomen with 600cc oral contrast
2. Tumor location / Size
Location:
Hepatic flexure
Size:
Measurable: 2.2 cm (largest diameter)
3. Tumor invasion
Tumor within colonic or rectal wall
4. Regional nodal metastasis
N1: Yes
location:
Middle colic
5. Distant metastasis (In this study)
No or Equivocal
6. Other findings:
> A 2.2cm enhancing lesion at hepatic flexure of colon(coronal:19), compatible with colon Ca.
> A 1.0cm mildly enlarged LN in the mesentary next to middle colic artery(Ser:4, img:46, coronal:10), metastatic lymphadenopathy can"t be R/O.
> Some mildly enlarged LNs at left upper paraaortic retroperitoneum. nature to be determined.
> R/O A 8.0mm hepatic cyst at S4 of liver(Ser:4, img:15).
> Mild DJD change of T-L spine with spur formation.
Impression:
R/O colon Ca. at hepatic flexure and metastatic lymphadenopathy next to middle colic artery can"t be R/O. Imaging stage T1-2N1M0.

特殊檢查

病理報告

其它

出院時情況

- 3.改門診治療

出院指示

藥名	劑量	劑量單位	用法	頻次	數量	數量單位	說況	天數	單位
Ultracet tab	1	TAB	PO	QID	20	TAB	TH	5	天
(□)Takepron OD tab 30mg	1	TAB	PO	QDAC	5	TAB	TH	5	天
Through tab	1	TAB	PO	QN	5	TAB	TH	5	天

出院計劃

主治醫師_2062F 蔣鋒帆_______________ / 住院醫師_2328K 邱俊凱_______________

正式摘要**(**電子簽章：**000000184299670)**

W52 / W52

doctorRank:B
